# Utilization of chemically treated cashew-nut shell as potential adsorbent for removal of Pb(II) ions from aqueous solution

**DOI:** 10.1038/s41598-020-60161-9

**Published:** 2020-02-24

**Authors:** Kamchai Nuithitikul, Rapeeporn Phromrak, Wikanda Saengngoen

**Affiliations:** 1 Department of Chemical Engineering, Walailak University, Nakhonsithammarat, 80160 Thailand; 2 Biomass and Oil Palm Center of Excellence, Walailak University, Nakhonsithammarat, 80160 Thailand

**Keywords:** Environmental sciences, Chemical engineering

## Abstract

In this study, cashew nut shells (CNS), waste from a cashew nut processing factory, have been used as an adsorbent for Pb(II) ions in water. Treatments of CNS with 1 M of H_2_SO_4_, HNO_3,_ and NaOH solutions were performed to modify their surfaces and improve their adsorption capacities. Characterization of untreated and chemical-treated CNS was carried out using nitrogen adsorption isotherm, elemental (CHN) analysis, Fourier-transform infrared spectroscopy (FTIR), and scanning electron microscopy (SEM) equipped with energy dispersive X-ray analysis (EDX). In the study of Pb(II) removal, various models of adsorption kinetics and isotherms were evaluated against the experimental data. The results showed that H_2_SO_4_-treated CNS exhibited the highest adsorption capacity. The chemical treatment removes impurities, alters the surface functional groups and improves specific surface areas and pore volumes of native CNS significantly. Surface adsorption and intra-particle diffusion steps were found to substantially affect the overall adsorption process of Pb(II) on H_2_SO_4_-treated CNS. Owing to its easy preparation and comparable adsorption capacity, H_2_SO_4_-treated CNS has the potential to be developed as a low-cost adsorbent for the removal of Pb(II) from contaminated water.

## Introduction

Water is a natural resource necessary for living and sustaining our ecosystem. The increases in human population and industrial processes have released more polluted water into the environment unless proper treatments are implemented effectively. Owing to the water solubility of heavy metals under favorable pH/Eh conditions, water is inevitably contaminated with heavy metals, mostly discharged from several industries^1^. Such contaminated water leads to environmental problems and accumulation of heavy metals in the food chain which finally returns to human beings. Numerous disorders and diseases are caused by the deposition of heavy metals. Ions of lead, mercury, cadmium and chromium have been reported to be at the top of the toxicity list^2^.

Lead (Pb) ions contaminated in water are highly toxic to humans and the environment. The sources of lead include the steel and metal alloy industries, welding and electroplating processes, and manufactures of batteries, pigments and ammunition^2–4^. The accumulation of lead in the human body at a certain level can seriously destroy the nervous system, organs (i.e., heart, kidneys, and reproductive system) and tissues^5,6^. The World Health Organization specifies the maximum concentration of lead ions in drinking water as 0.01 mg/L^7^.

Several methods have been introduced to remove heavy metal ions from contaminated water such as chemical precipitation, membrane filtration, ion-exchange, electrochemical methods, flotation, and adsorption. Many methods have drawbacks which are high capital and operating costs, the requirement of extra chemicals and high energy, generation of hazardous sludge, and low performance for diluted wastewater^8^. Among these methods, adsorption is very attractive owing to its easy operation and high efficiency to treat water contaminated with low concentrations of heavy metals, i.e., <100 mg/L^7^.

In the adsorption process, activated carbon has been widely recognized as an effective adsorbent for the removal of various pollutants contaminated in water. However, the industrial use of activated carbon is limited due to expensive manufacturing and regenerating processes^9^. To reduce the production and operation costs, there have been continuous attempts to develop low-cost adsorbents derived from industrial and agricultural wastes directly, e.g., cucumber peel^5^, rapeseed biomass^7^, cotton stalk and peanut hull^9^, olive tree pruning waste^10^, pine tree cone^11^, and cashew nut shell^12^. Biomass wastes are promising raw materials since they are increasingly generated.

A disadvantage of using biomass wastes as adsorbents directly is their limited adsorption capacities. To increase the adsorption capacity, chemical treatment (using H_2_SO_4_, HNO_3_, and NaOH) to modify the structure and surface of adsorbents has been adopted^13–16^.

The cashew nut processing industry has been growing, particularly in tropical countries such as Brazil, India, and Vietnam^17^. Cashew nut shell residues are mostly used as solid fuel in factories after extraction of cashew nut shell oil for industrial use. To utilize cashew nut shells efficiently and economically, several studies have been conducted, e.g., the development of cashew nut shells to activated carbons for the adsorption of methylene blue^18^ and fluoride^19^. Some researchers investigated the use of raw cashew nut shells as biosorbents for the adsorption of dyes^20,21^, nickel^22^, copper^23^, cadmium^24^, chromium^24^, and lead^12,24^. However, studies on chemical modification of cashew nut shells to improve the adsorption capacity are limited. In this study, therefore, cashew nut shells have been chemically modified, characterized and tested for the adsorption of Pb(II) ions. The aim is to determine and compare the adsorption capacities of chemical (H_2_SO_4_, HNO_3_ or NaOH)-treated cashew nut shells with untreated ones for removal of Pb(II) ions in water. Adsorption kinetics and isotherm were also determined from H_2_SO_4_-treated cashew nut shells which gave the greatest adsorption capacity for Pb(II) ions.

## Methods

### Preparation of cashew nut shell adsorbents

Cashew nut shells (CNS) were collected from a cashew nut processing factory to simply prepare adsorbents of Pb(II) ions. The CNS biomass was initially washed and dried at 105 °C before grinding to a smaller size (<2.0 mm). The ground solid was extracted with hexane to remove cashew nut shell liquid and the remaining shells were dried at 105 °C for 24 h. Portions of the shells were treated with aqueous solutions of H_2_SO_4_, HNO_3_ or NaOH (1 M each) at 30 °C for 24 h and subsequently washed with distilled water several times until the pH of washing water became constant, equal to the original value (pH = 6.9). These chemical-treated adsorbents were then dried at 105 °C for 1 h and kept in a desiccator before use.

During the chemical treatment of CNS, the percentage of weight loss was observed and determined. The weight loss is owing to the damaged structure of CNS, dissolution of some compositions, and operational loss from washing, filtering, and drying stages.

### Characterization of cashew nut shell adsorbents

Elemental analysis of untreated and chemical-treated CNS was performed with CHNS/O analyzer (Flash 2000, ThermoScientific, Italy). The C, H, N, and S contents were determined directly, whereas the oxygen content was calculated by the difference between unity and the sum of C, H, N, and S components. Brunauer-Emmett-Teller (BET) specific surface area, pore volume, and average pore diameter of untreated and chemical-treated CNS were determined based on N_2_ adsorption method at 77 K (ASAP2460, Micromeritics, USA). Before BET analysis, the samples were degassed at 80 °C for 5 h. The surface functional groups of untreated and chemical-treated CNS were determined using Fourier-transform infrared (FTIR) spectroscopy (TENSOR 27, Bruker) recorded in the region of 550–4000 cm^−1^ with a resolution of 2.0 cm^−1^. Scanning electron microscopy (SEM) equipped with energy dispersive X-ray analysis (EDX) was performed on Zeiss Merlin VP Compact operated at 2 kV. For each sample, images of the external and internal surfaces were taken at 1000x magnification.

### Adsorption study

Batch adsorption was carried out in 500 mL flasks. Typically, an aqueous solution of Pb(NO_3_)_2_ was prepared at the initial Pb(II) concentration of 50 mg/L. 2 g of untreated or chemical-treated CNS was added into the flasks. The adsorption temperature was kept constant at 30 ± 1 °C. The adsorption process was initiated by stirring at 500 rpm and continued for 24 h. During the experiment, samples were taken and rapidly centrifuged in order to separate the supernatant liquid from the solid adsorbent. The liquid samples were analyzed with an atomic absorption spectrometer (AAnalyst 800, Perkin Elmer) to determine the remaining concentrations of Pb(II). The adsorption capacities (the amount of Pb(II) adsorbed per unit mass of CNS, *q*_*t*_) were calculated from the initial concentration (*C*_0_) and remaining concentration (*C*_*t*_) of Pb(II) in the aqueous solutions as shown in Eq. (1) where *V* is the volume of solutions and *m* is the mass of CNS.

1 $${q}_{t}=\frac{({C}_{0}-{C}_{t})V}{m}$$

For adsorption kinetics and isotherm studies, the H_2_SO_4_-treated CNS which exhibited the highest adsorption capacity for Pb(II) was selected. The initial Pb(II) concentrations were varied between 10 and 50 mg/L. The experiments were conducted in a similar way as described above, and the data at the contact time up to 30 min was used to validate proposed kinetic models (pseudo-first order, pseudo-second order, Elovich, and intra-particle diffusion models). For the adsorption isotherm validation, the data at the equilibrium was used to fit with Langmuir, Freundlich, Temkin, and Dubinin-Radushkevich (D-R) isotherm models.

## Results and Discussion

### Characterization of CNS adsorbents

As shown in Table 1, treatments of CNS with acids (H_2_SO_4_, HNO_3_) and base (NaOH) solutions caused weight loss in the rage of 26.7–51.0%. The treatment with HNO_3_ gave the largest weight loss (51.0%) whereas the treatment with NaOH resulted in the smallest weight loss (26.7%). The weight loss after chemical treatment is due to the removal of remaining CNS oil and small particles attached to the surface of CNS. Moreover, the components of CNS fiber including its lignocellulosic contents (cellulose, hemicellulose, and lignin), could be hydrolyzed. Acids such as H_2_SO_4_ and HNO_3_ could dissolve hemicellulose components whereas bases such as NaOH promote the lignin destruction^25,26^. Raw cashew nut shells had crude fiber content of 23.05%, cellulose 11.50%, hemicellulose 7.35% and lignin 7.45%^27^. The oil composition in cashew nut shells was 8.30%^28^. Using hexane as an extracting solvent was not able to extract CNS oil completely. A previous study in the treatment of olive tree pruning with H_2_SO_4_, HNO_3_, and NaOH found similar weight loss in the range of 27.5–46.7%^10^.

**Table 1:** Properties of untreated CNS and chemical treated CNS.

Properties	Untreated CNS	H_2_SO_4_-treated CNS	HNO_3_-treated CNS	NaOH-treated CNS
*Weight loss (%)*	—	41.3	51.0	26.7
***Elemental analysis***				
C (%)	45.93	43.99	43.67	45.44
H (%)	5.75	5.75	6.36	5.83
N (%)	0.62	2.22	0.29	0.66
S (%)	0.13	0.09	0.01	0.12
O (%)^a^	47.57	47.95	49.67	47.95
O/C ratio	1.036	1.090	1.137	1.056
BET surface area (m^2^/g)	0.01	0.648	0.398	0.163
Pore volume (×10^4^ cm^3^/g)	ND^b^	12.19	8.62	0.16
Mean pore diameter (nm)	ND^b^	7.53	8.67	0.40

^a^By difference

^b^Not detectable.

Elemental analysis of untreated CNS and chemical-treated CNS gave the result as shown in Table 1. The C content of untreated CNS in this study was 45.93%, which is in agreement with the range (45.21–58.3%) reported in the previous literature^23,29–32^. The difference in the composition of CNS depends on the varieties and growing condition of cashew nut trees. It is important to note that oxidation could take place when CNS was treated with chemicals. As a result, the O/C ratios of all chemical-treated CNS were found to increase (with the decrease of C and increase of O contents) compared to untreated CNS. Chemical oxidation was reported to occur with HNO_3_ and H_2_SO_4_ treatments^33^.

BET specific surface area, pore volume, and average pore size of untreated CNS and chemical-treated CNS are summarized in Table 1. Chemical treatment significantly increased the specific surface area and pore volume of raw CNS. The acid treatment was found to improve the pore characteristics of CNS better than the base treatment. H_2_SO_4_-treated CNS exhibited the highest values of specific surface area (0.648 m^2^/g) and pore volume (12.19 ×10^–4^ cm^3^/g). NaOH-treated CNS gave the lowest values of specific surface area (0.163 m^2^/g) and pore volume (0.16 × 10^–4^ cm^3^/g). However, the specific surface areas of all chemically treated CNS are not high compared to those of commercial adsorbents.

**Figure 1:**
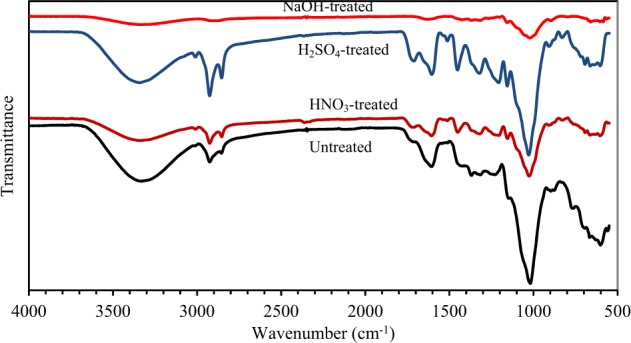
FTIR spectra of untreated and chemical (H_2_SO_4_, HNO_3_ or NaOH)-treated CNS.

FTIR spectra of untreated and chemical-treated CNS are shown in Fig. 1. The broad peaks at 3350–3330 cm^−1^ are assigned to -OH stretches of alcohols and phenols found in the lignocellulosic structure as well as absorbed water. HNO_3_- and NaOH-treated CNS had lower peak intensities of -OH groups than untreated and H_2_SO_4_-treated CNS. For untreated, H_2_SO_4_- and HNO_3_-treated CNS, the double peaks at ~2925 and ~2855 cm^−1^ could represent C-H stretching of CH_2_ and CH_3_ groups (asymmetric and symmetric stretches)^10^ and their bending vibration at ~1451 cm^−1^. These peaks were mostly noticed for H_2_SO_4_-treated CNS. The sharp peaks at ~1606 cm^−1^ could be assigned to C=C bonds^34^. The small peaks at 1711–1717 cm^−1^ in the spectra of untreated, H_2_SO_4_- and HNO_3_-treated CNS might be associated with C=O stretching of carboxylic acids, ketones, and aldehydes^7,34^.

**Figure 2:**
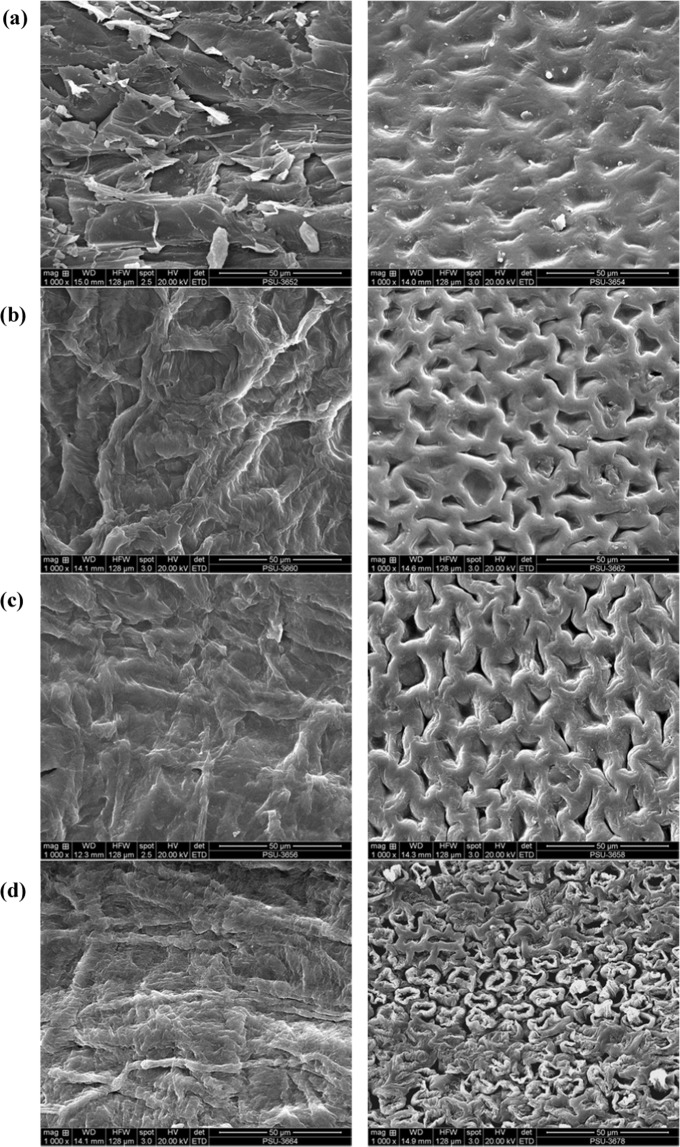
SEM images of (**a**) untreated; (**b**) H_2_SO_4_-treated; (**c**) HNO_3_-treated; and (**d**) NaOH-treated CNS (1000×).

In comparison to untreated CNS, sharper peaks were observed at 1028, 1155, and 1207 cm^−1^ for H_2_SO_4_-treated CNS (Fig. 1), suggesting an increase in functional groups with single oxygen bonds, e.g., ethers, esters, alcohols, phenols, and lactones. Peaks between 1000 and 1300 cm^−1^ indicated C-O stretching of these functional groups^13^. Similar to our result, the treatment of olive tree pruning waste with H_2_SO_4_ increased the peak intensity at 1000–1200 cm^−1^^10^. For H_2_SO_4_-treated CNS, sharper peaks were also found at 1321, 1451, 1606, and 1711 cm^−1^, compared to untreated and HNO_3_-treated CNS. The most prominent peaks of H_2_SO_4_-treated CNS are expected to play an important role in the increased sorption capacity for lead ions compared to untreated and other chemically-treated CNS as discussed later. For NaOH-treated CNS, intensities of peaks decreased significantly. The reduced intensities are probably due to delignification. Alkaline treatment has been widely reported to remove lignin content significantly^35^. FTIR spectra of pure lignin exhibited the peaks at ∼3400 cm^−1^ (-OH group in aromatic and aliphatic structures), 2850–2920 cm^−1^ (C-H bonds of CH_2_ and CH_3_ of propyl side chains), 1650–1720 cm^−1^ (C=O group), 1510–1600 cm^−1^ (C=C group in aromatic structure) and 700–1450 cm^−1^ (C-C and C-H bonds in aromatic structure)^36,37^. For NaOH-treated CNS, the intensities of peaks at these wavenumbers decreased significantly, confirming the removal of lignin components. Moreover, the collapse of CNS structure with NaOH treatment indicated the dissolution of lignin, as evidenced by the SEM image in Fig. 2d (right). Lignin is a major constituent providing strength to plant cell walls. Similar to our result, increasing the concentration of NaOH in the treatment process was reported to reduce peaks intensities in FTIR spectra^11^.

The lower peaks intensities of NaOH-treated CNS caused fewer numbers of active functional groups necessary for the binding of lead ions. As can be seen from Table 1, specific surface areas of CNS are improved with all chemical treatments but still low. Therefore, it is expected that the interaction between lead ions and surface functional groups of the adsorbent is more important than physical factors (i.e., specific surface area) for the removal of lead ions from water.

The external and internal surface structures of untreated and chemical (H_2_SO_4_, HNO_3,_ or NaOH)-treated CNS are shown in Fig. 2. Chemical treatment changes the surface texture of CNS. Untreated CNS has numerous irregular and small particles attached to both the external and internal surfaces. The treatment of CNS with all types of chemicals could wash out these particles from the surfaces, leaving smoother surfaces. The texture of CNS was damaged with HNO_3_ and H_2_SO_4_ treatments; as a result, significant shrinkage and more porous structure were noticed. These, in turn, generated a more specific surface area for the adsorption process. These results are in agreement with the result obtained from BET surface area analysis (Table 1). The treatment with NaOH also alters the texture of raw CNS but to a greater extent than the treatment with acids (Fig. 2). It is generally known that NaOH could break down the internal structure, particularly lignin of lignocellulosic materials. However, the number of voids in the structure of NaOH-treated CNS (Fig. 2d (left)) seems to be less than that of acid-treated CNS.

**Figure 3:**
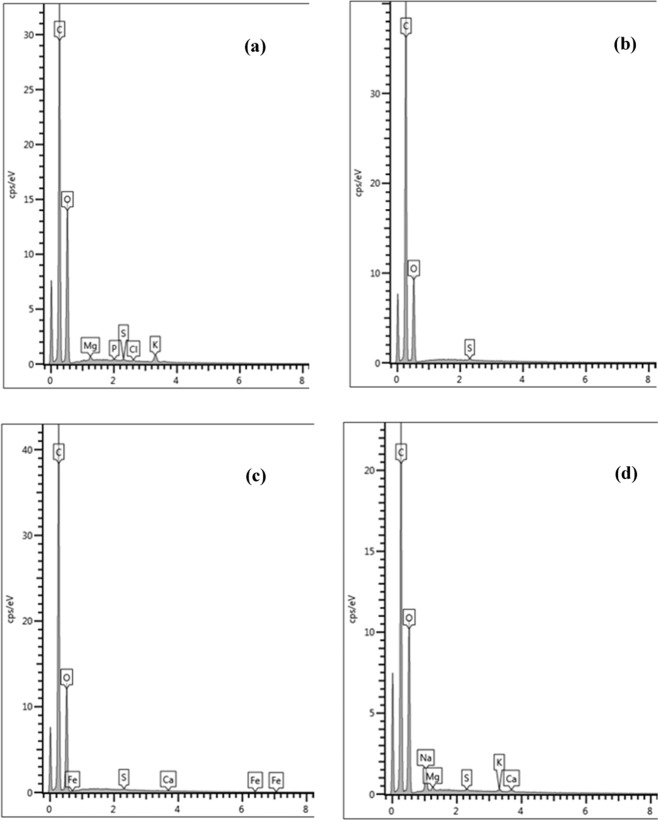
EDX spectra of (**a**) untreated; (**b**) H_2_SO_4_-treated; (**c**) HNO_3_-treated; and (**d**) NaOH-treated CNS.

EDX spectra of untreated and chemical (H_2_SO_4_, HNO_3,_ or NaOH)-treated CNS are presented in Fig. 3. Untreated CNS consisted of C, O, K, Mg, Cl, S and P elements. Treatment with H_2_SO_4_ significantly removed K, Mg, Cl, and P, leaving the surface with only C, O, and S. Similarly, treatment with a weaker acid (HNO_3_) removed K, Mg, Cl, and P. However, the presence of Fe and Ca was found owing to the intrinsic composition of CNS. The previous analysis of CNS ash showed the presence of silica, iron oxide, aluminium oxide, calcium oxide, and sodium oxide^22,23^. EDX spectra are linked to image maps generated by SEM, which is performed at a certain position on the surface of materials. When materials are heterogeneous, EDX analysis could give different results with various positions of SEM images. For NaOH treatment, the replacement of Cl and P with Na and Ca was noticed. Na might come from NaOH solution and intrinsic composition of CNS. Overall, elements were removed from the structure of CNS mostly by H_2_SO_4_, followed by HNO_3_ and NaOH treatments. Although the cationic elements (i.e., Na, K, Ca, and Mg) of the adsorbent were reported to increase lead adsorption^38^, it is not valid for this study. Other factors are influencing the adsorption of Pb(II) such as porosity, specific surface area and, most importantly, surface functional groups of the adsorbent.

### Adsorption study: effect of chemical treatment

**Figure 4:**
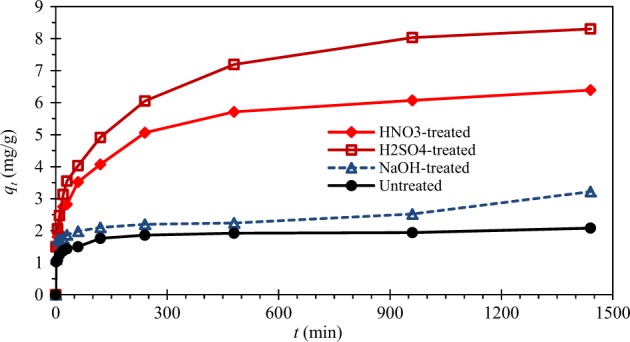
Effect of chemical treatment on Pb(II) adsorption [*C*_0_ = 50 mg/L].

The adsorption capacities of untreated and chemical (H_2_SO_4_, HNO_3,_ or NaOH)-treated CNS for Pb(II) are shown in Fig. 4. All the adsorption processes took place very quickly at the beginning (1 min of contact time) because many active sites are available for lead ions to adsorb. All chemical treatment could significantly improve the adsorption capacity of CNS. Adsorption equilibrium was attained after 24 h of contact time. The treatment of CNS with H_2_SO_4_ gave the highest adsorption capacity (8.30 mg/g), followed by those treated with HNO_3_ (6.39 mg/g) and NaOH (3.22 mg/g). The untreated CNS gave the equilibrium adsorption capacity only 2.08 mg/g.

The highest adsorption capacity of H_2_SO_4_-treated CNS could be related to the prominent C=O and C-O bonds found by FTIR analysis. The H_2_SO_4_ treatment was reported to alter the surface oxygen group species of activated carbon^39^. Moreover, the specific surface area and pore volume of H_2_SO_4_-treated CNS were the greatest among all chemically treated CNS (see Table 1).

HNO_3_-treated CNS gave higher lead adsorption capacity than NaOH-treated CNS. The higher adsorption capacity is probably due to the higher number of active functional groups as indicated by FTIR spectra. The specific surface area and pore volume of HNO_3_-treated CNS were also higher than NaOH-treated CNS (see Table 1). Therefore, it can be concluded that both chemical (i.e., surface functional groups) and physical (i.e., specific surface area and pore volume) factors are required for the adsorption capacity of lead ions on CNS.

NaOH-treated CNS gave higher lead adsorption capacity than untreated CNS although its FTIR spectrum exhibited lower intensity peaks. This result suggests that there are other factors than surface functional groups influencing the adsorption process. Physical factors (i.e., the removal of surface impurities and disintegration of fiber to generate more specific surface area) must be taken into account. Impurities attached to the surface or structure of CNS could inhibit the sorption of lead ions. As evidenced by EDX analysis (Fig. 3), H_2_SO_4_ treatment removes elements the best, followed by HNO_3_ and NaOH treatments. This is in agreement with the results of lead adsorption capacities (H_2_SO_4_ > HNO_3_ > NaOH). Hydrolysis reaction could take place with acid treatment more easily than base treatment, causing greater dissolution of organic substances and a severe disintegration of CNS fiber, as indicated by higher weight loss (Table 1). The H_2_SO_4_ treatment was previously found to improve the microporous surface area and volume of activated carbon^39^.

### Effect of the initial concentration of Pb(II)

**Figure 5:**
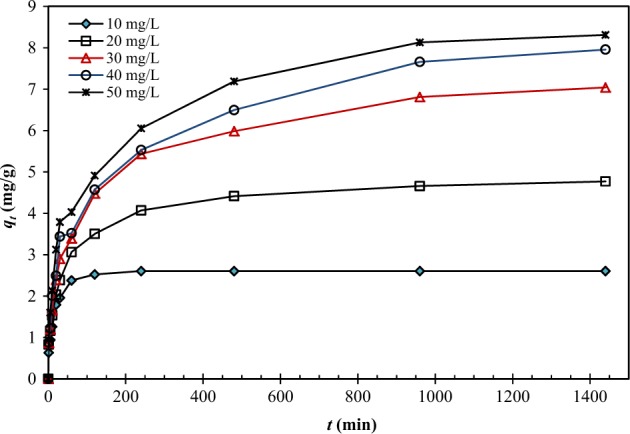
Effect of initial Pb(II) concentrations (*C*_0_ = 10–50 mg/L) on adsorption capacity of H_2_SO_4_-treated CNS.

Since H_2_SO_4_-treated CNS gave the highest adsorption capacity for Pb(II), it was selected as an adsorbent for further study in the adsorption kinetics and isotherm. The adsorption capacities of H_2_SO_4_-treated CNS were found to increase when the initial concentrations of Pb(II) solutions were increased from 10 to 50 mg/L (Fig. 5). However, the increased adsorption capacities were not linearly proportional to the initial concentrations, particularly at the high concentrations (40 and 50 mg/L). This is due to the limited surface area or active sites of the adsorbent. At low concentrations, there are sufficient active sites to adsorb Pb(II). The increased amount of Pb(II) could be supported by many unoccupied active sites. However, when the amount of Pb(II) in the solution is in excess, all Pb(II) species could not be adsorbed on the limited active sites of the adsorbent.

### Adsorption kinetics

Adsorption process involves mass transfer (both film and pore diffusions) and surface reaction (the attachment of adsorbate species on the active sites of adsorbent). Simple kinetic models, therefore, cannot be used to explain the process completely. In this study, pseudo-first order, pseudo-second order, Elovich, and intra-particle diffusion models were used to fit the experimental data to explain the adsorption process of Pb(II) on H_2_SO_4_-treated CNS.

#### Pseudo-first order model

The pseudo-first order model assumes that the adsorption capacity is directly proportional to the difference between the equilibrium concentration and concentration at any contact time^40^. In other words, the rate of occupation of adsorbent sites directly depends on the number of unoccupied sites^11^. The pseudo-first order equation was derived based on the assumption of physisorption control^41^. The linearized equation of pseudo-first order model is shown in Eq. (2).

2 $$log({q}_{e}-{q}_{t})=log({q}_{e})-\frac{{k}_{1}}{2.303}t$$

**Figure 6:**
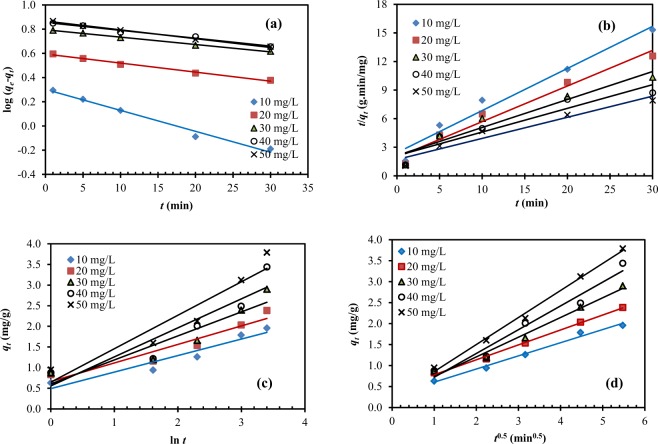
Kinetic plots for adsorption of Pb(II) on H_2_SO_4_-treated CNS at various initial concentrations, *C*_0_ = 10–50 mg/L: (**a**) pseudo-first order model, (**b**) pseudo-second order model, (**c**) Elovich model, (**d**) Intra-particle diffusion model.

*q*_*e*_ (mg/g) is the adsorption capacity at the equilibrium whereas *q*_*t*_ (mg/g) is the adsorption capacity at contact time *t* (min). *k*_1_ (min^−1^) is the first-order rate constant. The application of the pseudo-first order model to the experimental data is shown in Fig. 6(a) from which the values of *q*_e_ and *k*_1_ were determined from the intercepts and slopes of the plots (Table 2). The calculated values of *q*_e_ from pseudo-first order model were compared with those determined experimentally. The correlation coefficients (*R*^2^) were determined and found to be 0.9804–0.9963 for all initial concentrations of Pb(II). The *R*^2^ values indicate the validation of pseudo-first order model for explaining the adsorption of Pb(II) on H_2_SO_4_-treated CNS. The values of *k*_1_ were found to depend on the initial concentrations of Pb(II) at 10–20 mg/L; therefore, the adsorption is a surface reaction rate controlled process at low concentrations. However, the values of *k*_1_ decreased and were independent of the initial concentrations of Pb(II) at higher concentrations (30–50 mg/L). This suggests that mass transfer resistance becomes more important at higher concentrations.

**Table 2:** Regression coefficients and kinetic parameters of pseudo-first order, pseudo-second order, Elovich and intra-particle diffusion models.

Models	*C*_0_ (mg/L)				
	10	20	30	40	50
Experimental *q*_*e*_ (mg/g)	2.61	4.77	7.04	7.96	8.31
***Pseudo-first order***					
*k*_1_ (min^-1^)	0.040	0.017	0.014	0.015	0.017
Calculated *q*_*e*_ (mg/g)	2.01	3.93	6.23	7.16	7.35
*R*^2^	0.9832	0.9906	0.9963	0.9804	0.9944
***Pseudo-second order***					
*k*_2_ (g/mg/min)	0.080	0.072	0.040	0.029	0.029
*h* (mg/g/min)	0.411	0.513	0.468	0.473	0.584
Calculated *q*_*e*_ (mg/g)	2.26	2.68	3.40	4.03	4.51
*R*^2^	0.9692	0.9669	0.9343	0.9003	0.9407
***Elovich***					
*α* (mg/g.min^-1^)	1.368	1.963	1.636	1.554	1.799
*β* (g/mg)	2.501	2.224	1.716	1.421	1.233
*R*^2^	0.9186	0.9110	0.8670	0.8508	0.9019
***Intra-particle diffusion***					
*k*_int_ (mg/g/min^0.5^)	0.314	0.356	0.470	0.567	0.644
*C* (mg/g)	0.291	0.427	0.268	0.154	0.212
*R*^2^	0.9877	0.9959	0.9837	0.9659	0.9944

#### Pseudo-second order model

On the basis of the chemical adsorption process, the pseudo-second order model was developed^42^. The pseudo-second order model describes the initial attachment of adsorbates to form a monolayer, with the possible formation of other layers by physisorption^4^. The linearized equation of pseudo-second order model is shown in Eq. (3) in which *k*_2_ (g/mg/min) is the rate constant of pseudo-second order. The initial adsorption rate, *h* (mg/g/min), is defined in Eq. (4).

3 $$\frac{t}{{q}_{t}}=\frac{1}{{k}_{2}{q}_{e}^{2}}+\frac{1}{{q}_{e}}t$$

4 $$h={k}_{2}{q}_{e}^{2}$$

The application of the pseudo-second order model to the experimental data is shown in Fig. 6(b) from which the values of *q*_e_, *k*_2_ and *h* were determined from the intercepts and slopes of the plots (Table 2). The *R*^2^ values (0.9003–0.9692) indicate that the pseudo-second order model provides a reasonable fit to the experimental data. However, in comparison to the pseudo-first order model, the *R*^2^ values were lower. In addition, a larger difference in *q*_e_ values determined from the pseudo-second order model and experiment were noticed. Therefore, the adsorption of Pb(II) onto H_2_SO_4_-treated CNS obeys the Lagergren pseudo-first order kinetic model.

#### Elovich model

The Elovich model explaining the chemisorption process for the heterogeneous system was first applied to the gas-solid system^43,44^. The linearized equation of the Elovich model is shown in Eq. (5). In this model, *α* (mg/g/min) represents the initial adsorption rate constant whereas *β* (g/mg) represents the desorption constant concerning the surface coverage and the activation energy of the chemisorption process. The application of the Elovich model to the experimental data is shown in Fig. 6(c) from which the values of *α* and *β* were determined from the intercepts and slopes. The low *R*^2^ values (Table 2) imply the invalidity of the Elovich model for the adsorption of Pb(II) on H_2_SO_4_-treated CNS.

5 $${q}_{t}=\frac{1}{\beta }ln(\alpha \beta )+\frac{1}{\beta }ln(t)$$

#### Intra-particle diffusion model

As shown in Eq. (6), the intra-particle diffusion model^45^ is typically used to explain the importance of the diffusion process of adsorbate molecules into the porous structure of adsorbent. *k*_int_ (mg/g.min^−0.5^) is the intra-particle rate constant and *C* (mg/g) is related to the boundary layer thickness. Both values were determined from the slopes and intercepts of the plots between *q*_*t*_ and *t*^1/2^ as shown in Fig. 6(d). As shown in Table 2, the high *R*^2^ values (>0.95) show a good fit, suggesting the suitability of this model and the importance of intra-particle diffusion. However, the intercepts of the plots do not pass through the origin (Fig. 6d) suggesting that intra-particle diffusion is not the sole rate-limiting step^46^ for the adsorption of Pb(II) on H_2_SO_4_-treated CNS. The intra-particle diffusion solely controls the overall adsorption process only when the intercept of the plot between *q*_*t*_ and *t*^1/2^ is zero^34^. Moreover, when the intercept becomes higher, the boundary layer effect is more important^6,47^.

6 $${q}_{t}={k}_{int}{t}^{1/2}+C$$

The *R*^2^ values and kinetic parameters, according to the models described above are summarized in Table 2. Overall, the *R*^2^ values of the pseudo-first order are higher than those of the pseudo-second order model. The calculated values of *q*_e_ from the pseudo-first order model were closer to the experimental values than the pseudo-second order model. The pseudo-first order model was derived based on the assumption of physisorption control^41^. The *R*^2^ values of the Elovich model, which explains chemisorption process were found to be lower than those obtained from the pseudo-first and pseudo-second order models. These imply that the adsorption of Pb(II) onto the surface of H_2_SO_4_-treated CNS is rather physisorption than chemisorption process. Moreover, the *R*^2^ values of the intra-particle diffusion model were high (>0.98) at many initial concentrations of Pb(II), which suggests that intra-particle diffusion is as essential as surface adsorption (which is physisorption rather than chemisorption) and cannot be neglected.

### Adsorption isotherms

The adsorption equilibrium data obtained from varying the initial concentrations of Pb(II) were analyzed according to Langmuir, Freundlich, Temkin, and Dubinin-Radushkevich isotherms.

#### Langmuir isotherm

**Figure 7:**
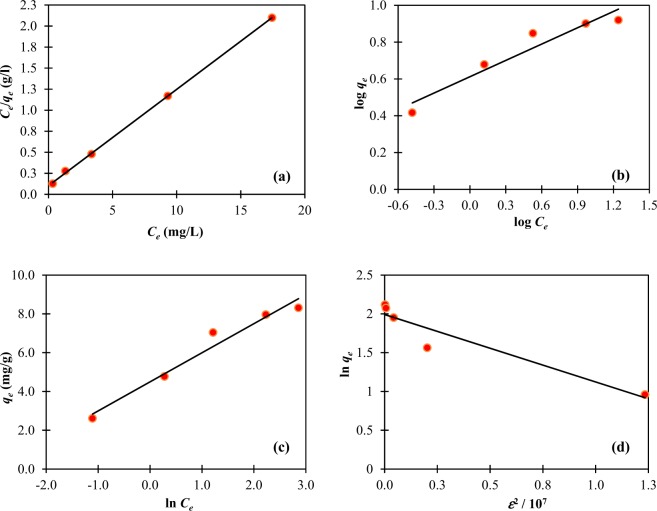
Isotherm plots for adsorption of Pb(II) on H_2_SO_4_-treated CNS at various initial concentrations, *C*_0_ = 10–50 mg/L: (**a**) Langmuir isotherm, (**b**) Freundlich isotherm, (**c**) Temkin isotherm, (**d**) Dubinin-Radushkevich isotherm.

The Langmuir isotherm describes the ideal situation in which the attachment of adsorbates onto a homogenous surface of the adsorbent is monolayer without interaction between the adsorbates^48^. The adsorption energy of each adsorbate is identical and independent of the adsorbent surface. The linearized form of the Langmuir isotherm is shown in Eq. (7) in which *q*_*max*_ (mg/g) and *b* (l/mg) represent the monolayer (maximum) adsorption capacity and energy of adsorption, respectively. Both values were determined from the slope and intercept of the plot between *C*_*e*_ and *C*_*e*_*/q*_*e*_ (Fig. 7a). A perfect fit of the Langmuir isotherm with the high value of *R*^2^ (0.9997) was obtained. The values of *q*_*max*_ and *b* were 8.734 mg/g and 1.115 L/mg as reported in Table 3. As indicated by the higher value of *b*, lead ions had stronger interaction with the surface of H_2_SO_4_-treated CNS than that of lobeira fruit (*b* = 0.02 L/mg)^3^.

7 $$\frac{{C}_{e}}{{q}_{e}}=\frac{1}{{q}_{max}b}+\frac{{C}_{e}}{{q}_{max}}$$

**Table 3:** Regression coefficients and parameters of Langmuir, Freundlich, Temkin and Dubinin-Radushkevich isotherms.

Isotherms	Constants
***Langmuir***	
*q*_*max*_ (mg/g)	8.734
*b* (L/mg)	1.115
*R*^2^	0.9997
***Freundlich***	
*K*_*F*_ (mg/g).(L/mg)^1/*n*^	4.094
*n*	3.385
*R*^2^	0.9229
***Temkin***	
*B*	1.499
*b* (kJ/mol)	1.681
*A*_*T*_ (L/mg)	20.087
*R*^2^	0.9641
***Dubinin-Radushkevich***	
*β* (mol^2^/J^2^)	9 ×10^–8^
*q*_*s*_ (mg/g)	7.33
*E* (kJ/mol)	2.36
*R*^2^	0.9041

**Figure 8:**
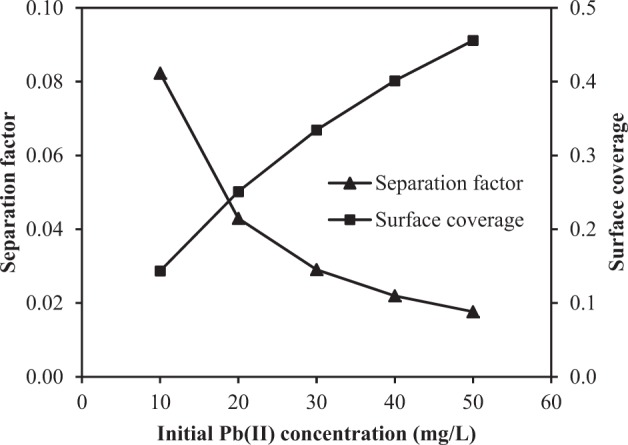
A plot of separation factor and surface coverage against initial concentrations of Pb(II).

An important parameter of the Langmuir isotherm is called separation factor (*R*_*L*_) determined from Eq. (8), in which *C*_0_ is the initial concentration of Pb(II). The value of *R*_*L*_ could indicate the shape of isotherm and nature of the adsorption process: favorable isotherm (*0* < *R*_*L*_ < 1), irreversible isotherm (*R*_*L*_ = 0), linear isotherm (*R*_*L*_ = 1), and unfavorable isotherm (*R*_*L*_ > 1). The closer the *R*_*L*_ value is to 0, the more favorable is the adsorption process^49^. In Fig. 8, the values of *R*_*L*_ were found to be between 0 and 0.1 at all the initial concentrations of Pb(II) indicating that the adsorption of Pb(II) on H_2_SO_4_-treated CNS is favorable. Moreover, when the initial concentration of Pb(II) was increased, the adsorption became more favorable and irreversible, as indicated by the lower values of *R*_*L*_.

8 $${R}_{L}=\frac{1}{1+b{C}_{0}}$$

According to the Langmuir isotherm, the adsorption behavior of Pb(II) on H_2_SO_4_-treated CNS is related to surface coverage (*θ*) of the adsorbent, as shown in Eq. (9) where *K* is equilibrium adsorption constant. The plot of the surface coverage with the initial concentrations of Pb(II) is shown in Fig. 8. When the initial concentration was increased, the surface of the H_2_SO_4_-treated CNS was more covered with the attached atoms of Pb(II). At the initial concentration of Pb(II) of 50 mg/L, the surface coverage was 0.46, which means that the surface still has vacant sites for Pb(II) to adsorb further. However, the adsorption rate tends to decrease at the initial concentration higher than 50 mg/L since the relationship between surface coverage and initial concentration begins to level off.

9 $$K{C}_{0}=\frac{\theta }{1-\theta }$$

#### Freundlich isotherm

The Freundlich isotherm describes the attachment of adsorbate species onto a heterogeneous surface of the adsorbent being either monolayer or multilayer with an interaction between the adsorbate species^50^. The linearized form of the Freundlich model is presented in Eq. (10). The *K*_*F*_ constant indicates the adsorption capacity, while *n* implies the adsorption effectiveness: favorable adsorption (1 < *n* < 10)^51^. Both constants were determined from the intercept and slope of the plot between log *q*_*e*_ and log *C*_*e*_ (Fig. 7b). As shown in Table 3, the *K*_*F*_ and *n* values were 4.094 (mg/g)(L/mg)^1/*n*^ and 3.385, respectively. The value of *n* is higher than unity suggesting easy separation and favorable adsorption of Pb(II) on H_2_SO_4_-treated CNS. The *R*^2^ value using the Freundlich model was found to be lower than 0.95, suggesting that the adsorption surface of H_2_SO_4_-treated CNS deviated from the heterogeneous surface. Owing to the very high value of *R*^2^ using the Langmuir isotherm, it could be interpreted that the surface of H_2_SO_4_-treated CNS was rather homogeneous.

10 $$log({q}_{e})=log({K}_{F})+(1/n)log({C}_{e})$$

#### Temkin isotherm

The adsorbate-adsorbent interaction is taken into account when the Temkin isotherm is used to explain the adsorption process. Such interaction causes a linear decrease in the heat of adsorption of all molecules in the layer with the increasing coverage^3^. The Temkin model is different from the Freundlich model in which the reduction in the heat of adsorption is linear, not logarithmic, as reported in the Freundlich isotherm^5,22^. The linearized form of the Temkin isotherm is shown in Eq. (11) where *B* = *RT*/*b* is a constant related to the heat of adsorption, *R* is the gas constant (8.314 J/mol/K), and *A*_*T*_ (L/mg) is a Temkin binding constant. The values of *A*_*T*_ (20.087 L/mg) and *b* (1.681 kJ/mol) were determined from the intercept and slope of the plot between *q*_*e*_ and ln *C*_*e*_ (Fig. 7c). When the bonding energy value is low, e.g., <8 kJ/mol, weak adsorbate-adsorbent interaction is formed and the adsorption mechanism mainly involves the physical adsorption^3^. Based on the value of *b* (1.681 kJ/mol), it can be concluded that the adsorption of Pb(II) on H_2_SO_4_-treated CNS is rather a physisorption. The result is in agreement with the value of *n* (*n* > 1), suggested by the Freundlich equation. Moreover, the heat of adsorption of Pb(II) on various adsorbents was previously found to be low, e.g., adsorption of Pb(II) onto banana peels^52^.

11 $${q}_{e}=Bln\,({A}_{T})+Bln({C}_{e})$$

#### Dubinin-Radushkevich (D-R) isotherm

The Dubinin-Radushkevich isotherm is used to explain the adsorption process on a heterogeneous and porous surface with variable parameters^34^. The linearized form of the Dubinin-Radushkevich isotherm is shown in Eq. (12) where *q*_*s*_ (mg/g) is theoretical saturation capacity, *β* (mol^2^/J^2^) is a constant related to adsorption energy, and *ε* is the Polanyi potential relating to the equilibrium concentration as shown in Eq. (13). The plot between ln *q*_*e*_ and *ε*^2^ is shown in Fig. 7(d) from which *q*_*s*_ (7.33 mg/g) and *β* (9×10^–8^ mol^2^/J^2^) were determined from the intercept and slope. The constant *β* is used to calculate the mean free energy (*E*) of adsorption of adsorbates based on Eq. (14). The value of *E* can indicate the type of adsorption process: physical adsorption (*E* < 8 kJ/mol) and chemical adsorption (*E* > 8 kJ/mol)^9,51^. The value of *E* in this study was found to be 2.36 kJ/mol, suggesting that the adsorption of Pb(II) on H_2_SO_4_-treated CNS is physisorption. This is in agreement with the result obtained from the application of the Temkin model to the experimental data.

12 $$ln\,({q}_{e})=ln\,({q}_{s})-\beta {\varepsilon }^{2}$$

13 $$\varepsilon =RTln\,(1+\frac{1}{{C}_{e}})$$

14 $$E=\frac{1}{\sqrt{2\beta }}$$

As summarized in Table 3, the Langmuir isotherm gave the best fit to the experimental data compared to other isotherms. The highest *R*^2^ value (0.9997) was obtained. This implies that the adsorption of Pb(II) on the homogeneous surface of H_2_SO_4_-treated CNS is monolayer and favorable.

According to the Langmuir isotherm, the maximum adsorption capacity of Pb(II) on H_2_SO_4_-treated CNS was 8.734 mg/g. The comparison in the maximum adsorption capacity of this study with previous researches is summarized in Table 4. Since the adsorption conditions are not identical, direct comparison could not be made. However, the maximum adsorption capacity of H_2_SO_4_-treated CNS is comparable to that of various biosorbents reported in the earlier literature. Under similar conditions (adsorbent loading, Pb(II) concentration, and temperature), the *q*_max_ value of H_2_SO_4_-treated CNS was of the same order of magnitude as of biosorbents derived from agricultural wastes such as cedar leaf ash^53^, peanut shell^54^, pomelo peel^55^, soya bean seed^56^, and mushroom biomass^57^. At the same adsorbent loading (4 g/L), H_2_SO_4_-treated CNS gave higher value of *q*_max_ than the adsorbents derived from plum and apricot kernels^1^. When the kernels were further developed to biochars by thermochemical process, the values of *q*_max_ increased significantly. However, such process is complicated, consumes extra energy, and requires further investment. Another crucial factor affecting the value of *q*_max_ is adsorption temperature. Adsorption capacity typically decreases with increasing temperature. As shown in Table 4, Pb(II) adsorption by H_2_SO_4_-treated CNS was carried out at higher temperature than most adsorbents. At temperatures below 30 °C; therefore, higher values of *q*_max_ would be obtained for H_2_SO_4_-treated CNS. Based on the comparable adsorption capacity with various biosorbents, H_2_SO_4_-treated CNS has the potential to be practically used for the treatment of Pb(II) in contaminated water.

**Table 4:** Comparison of maximum adsorption capacity (*q*_max_) for Pb(II) of this study with previous researches.

Adsorbent	Adsorption conditions			*q*_max_ (mg/g)	References
	Pb(II) concentration (mg/L)	Adsorbent loading (g/L)	Temperature (°C)		
H_2_SO_4_-treated CNS	10–50	4	30	8.73	This study
Apricot kernel	5–300	4	22	0.9	^1^
Plum kernel	5–300	4	22	1.3	^1^
Apricot-kernel biochar	5–300	4	22	23.9	^1^
Plum-kernel biochar	5–300	4	22	28.8	^1^
Cedar leaf ash	2–50	10	20	7.23	^53^
Peanut shell	N/A	20	30	7.13	^54^
Pomelo peel	10–30	10	30	2.139	^55^
Soya bean seed	1240	30	28	0.72	^56^
Mushroom biomass	25–1000	2.4	Room	3.89	^57^

## Conclusions

Chemical treatment of CNS significantly improves its adsorption capacity for Pb(II) in water. Chemical treatment not only alters the functional groups but also eliminates impurities attached to the surface or structure of CNS. Moreover, the porous structure of the raw CNS is significantly improved. As a result, specific surface area and pore volume are increased.

Different chemicals give adsorbents with varying adsorption capacities for Pb(II). H_2_SO_4_-treated CNS exhibited the highest adsorption capacity, followed by HNO_3_-treated CNS and NaOH-treated CNS. Acids treatment was found to generate better adsorbents than base treatment in terms of surface functional groups and specific surface area.

Pseudo-first order and intra-particle diffusion models explained the adsorption kinetics of Pb(II) on H_2_SO_4_-treated CNS very well. This suggests the importance of intra-particle diffusion step as well as the surface adsorption. The adsorption isotherm was best described with the Langmuir model. Since H_2_SO_4_-treated CNS could be prepared easily and its maximum adsorption capacity was comparable to various biosorbents reported in the previous literature, it is, therefore, a promising low-cost adsorbent for removal of Pb(II) from contaminated water.
